# PARP6 acts as a tumor suppressor via downregulating Survivin expression in colorectal cancer

**DOI:** 10.18632/oncotarget.7712

**Published:** 2016-02-25

**Authors:** Guangying Qi, Yasusei Kudo, Bo Tang, Tian Liu, Shengjian Jin, Jing Liu, Xiaoxu Zuo, Sisi Mi, Wenhuan Shao, Xiaojuan Ma, Takaaki Tsunematsu, Naozumi Ishimaru, Sien Zeng, Masaaki Tatsuka, Fumio Shimamoto

**Affiliations:** ^1^ Department of Pathology and Physiopathology, Guilin Medical University, Guilin 541004, People's Republic of China; ^2^ Department of Health Sciences, Prefectural University of Hiroshima, Hiroshima 734-8558, Japan; ^3^ Department of Oral Molecular Pathology, Institute of Biomedical Sciences, Tokushima University Graduate School, Tokushima 770-8504, Japan; ^4^ Department of Hepatobiliary Surgery, Affiliated Hospital of Guilin Medical University, Guilin 541000, People's Republic of China; ^5^ Department of Life Sciences, Faculty of Life and Environmental Sciences, Prefectural University of Hiroshima, Hiroshima 727-0023, Japan

**Keywords:** PARP6, Survivin, colorectal cancer, tumor suppressor, prognosis

## Abstract

Poly (ADP-ribose) polymerases (PARPs) are enzymes that transfer ADP-ribose groups to target proteins and are involved in a variety of biological processes. PARP6 is a novel member, and our previous findings suggest that PARP6 may act as a tumor suppressor via suppressing cell cycle progression. However, it is still unclear that PARP6 function besides growth suppression in colorectal cancer (CRC). In this study, we examined tumor suppressive roles of PAPR6 in CRC cells both *in vitro* and *in vivo*. We found that PARP6 inhibited colony formation, invasion and migration as well as cell proliferation. Moreover, ectopic overexpression of PARP6 decreased Survivin expression, which acts as an oncogene and is involved in apoptosis and mitosis. We confirmed the inverse correlation between PARP6 and Survivin expression in CRC cases by immunohistochemistry. Importantly, CRC cases with downregulation of PARP6 and upregulation of Survivin showed poor prognosis. In summary, PARP6 acts as a tumor suppressor via downregulating Survivin expression in CRC. PARP6 can be a novel diagnostic and therapeutic target together with Survivin for CRC.

## INTRODUCTION

It is well accepted that colorectal cancer (CRC) is the third most common type of cancer and a major cause of cancer-related mortality worldwide [1, 2]. At the same time, this global public health problem places a major economic burden on the global health care system, particularly in developing regions. Thus, there is an increased need for early diagnosis, drug response predictions and other clinical applications associated with CRC.

The presence of Poly (ADP-ribosyl) ation activity was originally suggested in the 1960s [3, 4]. PARPs are multifunctional nuclear proteases that mediate post-translational protein modification in the process of poly-ADP-glycosylation [5, 6]. Post-translational modification of proteins by poly (ADP-ribosyl) ation is involved in a variety of biological process including DNA repair, chromatin structural regulation, gene transcription, cell cycle progression and cell death [5, 6]. At present, 17 members of the PARP family have been identified, such as PARP1-4, 5α, 5β and 6-16 [7, 8]. Moreover, the PARP family is correlated with the development of various diseases [9, 10]. Among the PARPs, PARP-1, which produces polymers of ADP-ribose (PAR) using NAD+ as a substrate, is well studied. PARP-1 promotes tumor angiogenesis [7, 11, 12], and elevated expression of PARP-1 is found in various cancers including breast [13], prostate [14], pancreatic [15] and gastric cancer [16].

PARP6, a new member of the PARP family, is located on chromosome 15q23. PARP6 contains the catalytic domain of PARP with a considerably shorter nicotinamide-ribose-binding site than PARP1 and belongs to the mono (ADP-ribosyl) transferase class [11, 12, 17]. We previously showed that ectopic overexpresssion of PARP6 in HeLa cells induced growth suppression *in vitro* [17]. Moreover, PARP6 positivity negatively correlated with the Ki-67 proliferation index and better prognosis in CRC [17]. Our previous findings suggest that PARP6 may act as a tumor suppressor via suppressing cell cycle progression. However, it is still unclear that PARP6 function besides growth suppression in the development of CRC.

Survivin is a member of the inhibitor of apoptosis protein (IAP) family, which participates in the inhibition of apoptosis, and its overexpression is associated with a poor outcome in a variety of human cancers [18–20]. We have previously shown that Survivin overexpression is correlated with malignant behavior of CRC, hepatocellular carcinoma and oral squamous cell carcinoma [21–26]. Moreover, we found possible correlation between Survivin and cell proliferation activity in these cancers. Supportively, besides inhibition of apoptosis, Survivin regulates chromosome segregation and cytokinesis as a chromosomal passenger protein (CPC) that forms a complex with Aurora-B, INCENP and Borealin [27, 28].

Here we examined PARP6 expression and its correlation with Survivin in a large number of CRC cases. Moreover, we examined the tumor suppressive function of PARP6 in CRC cells both *in vitro* and *in vivo*.

## RESULTS

### PARP6 inhibits cell proliferation and promotes apoptosis in CRC cells

**Figure 1:**
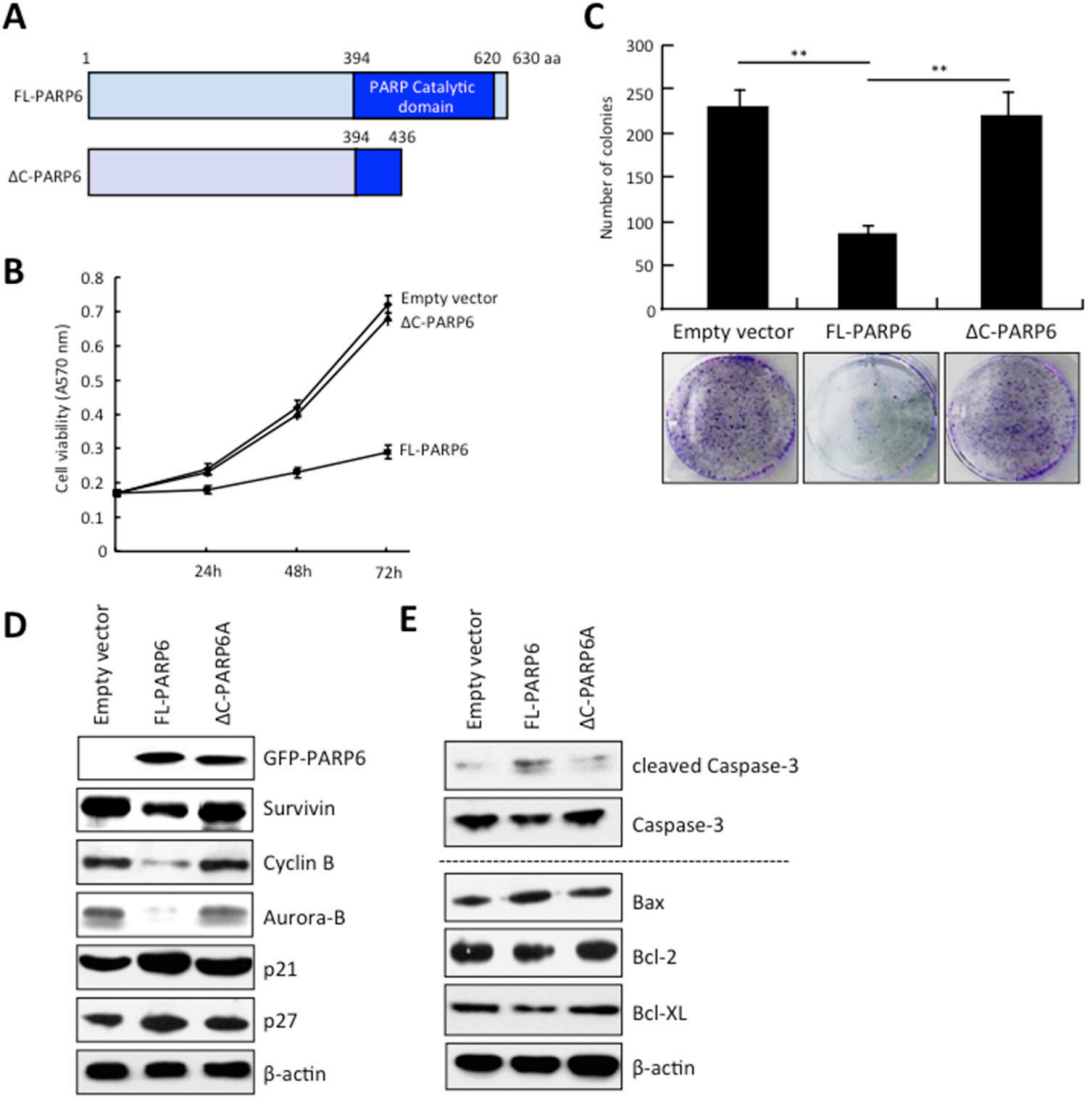
PARP6 inhibits cell growth and colony formation **A.** A schematic illustration of the human PARP6 protein. PARP6 consists of 630 amino acid residues and has a PARP catalytic domain in C-terminal region. ΔC-PARP6 lacks the PARP catalytic domain. **B.** Cell proliferation after FL-PARP6 and ΔC-PARP6 overexpression in SW480 cells was measured by MTT assays. After transfection with the p-EGFP-empty, p-EGFP-FL-PARP6 or p-EGFP-ΔC-PARP6 plasmids, transfectant cells (1500 cells / well) were replated in 96-well plates. The MTT assay was performed to test the cell viability at 24 h, 48 h and 72 h. Values indicate mean ± SD (n=6). **C.** Colony formation aof FL-PARP6 and ΔC-PARP6 transfectant SW480 cells. Cells were plated in 6 cm dishes at a density of 500 cells per dish. After 2 weeks, number of colonies were counted. The data represent the means ± S.D. of three independent experiments **P < 0.01. **D.** Expression of cell cycle related proteins including Survivin, Cyclin B, Aurora-B, p21 and p27 in empty vector, FL-PARP6 and ΔC-PARP6 transfectant SW480 cells was examined by Western blot analysis. β-actin expression was used as a loading control. **E.** Expression of apoptosis related proteins including cleaved Caspase-3, Caspase-3, Bax, Bcl-2 and Bcl-XL in empty vector, FL-PARP6 and ΔC-PARP6 transfectant SW480 cells was examined by Western blot analysis. β-actin expression was used as a loading control.

To know the tumor suppressive roles of PAPR6 in CRC cells, we used a full-length PARP6 (FL-PARP6) and C-terminal deletion mutant PARP6 (ΔC-PARP6). FL-PARP6 consists of 630 amino acids and the C-terminal region (residues 394–620) contains the PARP catalytic domain (Figure 1A). ΔC-PARP6 lacks the critical structure of the PARP catalytic domain. The SW480 cells were transfected with the empty, FL-PARP6 and ΔC-PARP6 vectors. FFL-PARP6, but not ΔC-PARP6 and control inhibited cell growth (Figure 1B). Moreover, FL-PARP6 significantly reduced the colony formation activity of SW480 cells, in comparison with control and ΔC-PARP6 (Figure 1C). We examined the expression of cell cycle related proteins including Survivin, Cyclin B, Aurora-B, p21 and p27. Interestingly, ectopic overexpression of FL-PARP6 decreased the expression of Survivin, Cyclin B and Aurora-B and increased the expression of p21 and p27, indicating that PARP6 inhibited cell growth via suppressing cell cycle progression (Figure 1D). Overexpression of ΔC-PARP6 had no effect on the expression of these proteins (Figure 1D).

Survivin is a CPC protein and controls chromosome segregation and cytokinesis [27]. In addition, Survivin is well known as a member of the inhibitor of apoptosis protein (IAP) family [18]. Here we found the downregulation of Survivin by overexpression of FL-PARP6 (Figure 1D). Moreover, we observed dead cells after overexpression of FL-PARP6 in CRC cells (data not shown). Indeed, we observed accumulation of cleaved Caspase-3 protein in FL-PARP6 transfectant cells, but not in ΔC-PARP6 and empty vector transfectant cells (Figure 1E). Moreover, we examined the expression of apoptosis related proteins including Bax, Bcl-2 and Bcl-XL in PARP6 transfectant CRC cells. We observed that decreased expression of apoptosis-promoting protein (Bax) and increased expression of apoptosis-inhibiting proteins (Bcl-2 and Bcl-XL) were observed in FL-PARP6 transfectant cells, but not ΔC-PARP6 and empty vector transfectant cells (Figure 1E).

### PARP6 inhibits migration and invasion of CRC cells

**Figure 2:**
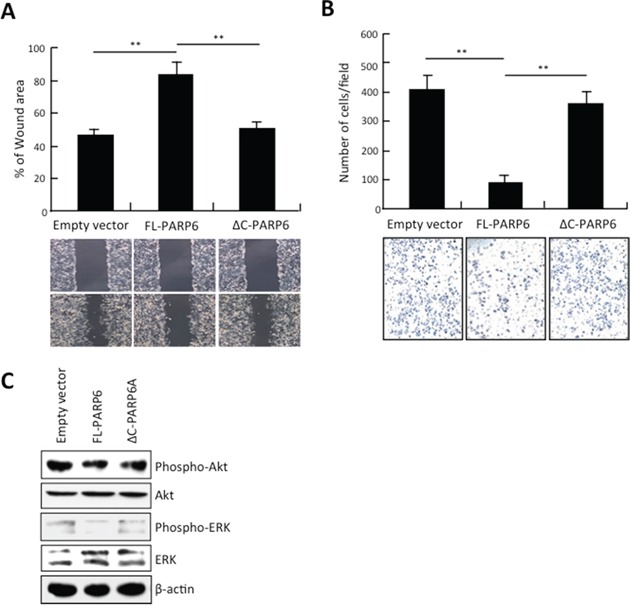
PARP6 inhibits invasion and migration **A.** Empty vector, FL-PARP6 and ΔC-PARP6 transfectant cells were subjected to wound healing assays. The uncovered areas as a percentage of the original wound area were quantified. **, *P* < 0.01 is based on the Student *t* test. All results were from three independent experiments. Error bars, SD. **B.** Empty vector, FL-PARP6 and ΔC-PARP6 transfectant cells were subjected to *in vitro* invasion assays using matrigel. Number of cells that invaded through matrigel was counted. We performed the assay 3 times and 5 randomly selected fields from each membrane were counted under a light microscope at x100 magnification. **, *P*< 0.01 is based on the Student *t* test. Error bars, SD. **C.** Expression of phospho- and total protein of Akt and ERK in empty vector, FL-PARP6 and ΔC-PARP6 transfectant SW480 cells by Western blot analysis. β-actin expression was used as a loading control.

Next, we examined the effect of ectopic overexpression of PARP6 in CRC cells on migration and invasion by wound healing assay and *in vitro* invasion assay, respectively. CRC cells expressing FL-PARP6 exhibited a significantly larger wound area compared to ΔC-PARP6 or empty vector transfectant cells (Figure 2A). FL-PARP6 dramatically inhibited cell invasion, while ΔC-PARP6 and empty vector showed a greater degree of invasion (Figure 2B).

We examined the activity of Akt and ERK, which are correlated with cell growth, migration, invasion and apoptosis. As shown in Figure 2C, the levels of phospho-Akt and phospho-ERK were significantly decreased in FL-PARP6 overexpressing CRC cells. However, ΔC-PARP6 overexpression had no effect on the expression of phospho-Akt and phospho-ERK. These findings suggest that tumor suppressive function of PARP6 may be mediated by Akt and/or ERK signaling pathway in CRC.

### PARP6 inhibits tumor growth *in vivo*

**Figure 3:**
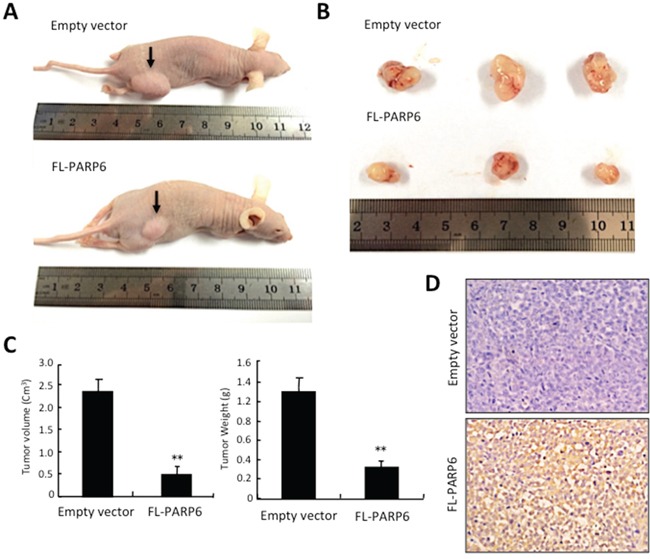
PARP6 inhibits tumor formation *in vivo* **A.** Empty vector and FL-PARP6 transfectant cells were subcutaneously inoculated into the lower right flank of the nude mice. **B.** Representative images of empty vector and FL-PARP6 transfectant tumors following subcutaneous injection. **C.** The tumor volume was calculated using the following formula: tumor volume (mm^3^) =0.5 × length (mm) × width^2^ (mm^2^). The tumor weight was measured. Left graph shows tumor volume, and right graph shows tumor weight in empty vector and FL-PARP6 transfectant tumors. **P < 0.01. **D.** Expression of PARP6 protein in empty vector and FL-PARP6 transfectant tumors was examined by immunohistochemistry.

We examined the tumor suppressive role of PARP6 *in vivo*. We subcutaneously transplanted FL-PARP6 or empty vector transfected CRC cells into the right groin area of the nude mice. After 28 days of transplantation, the tumors in FL-PARP6 transfectant group were consistently smaller than those in empty vector transfectant group (Figure 3A and 3B). Moreover, the sizes and weights of the tumors at 28 days after transplantation were significantly lower in FL-PARP6 transfectant group, in comparison with empty vector transfectant group (Figure 3C). We confirmed that high levels of PARP6 expression was observed only in FL-PARP6 transfectant group, but not in empty vector transfectant group (Figure 3D).

### PARP6 expression is inversely correlated with the Ki-67 and Survivin in CRC

**Figure 4:**
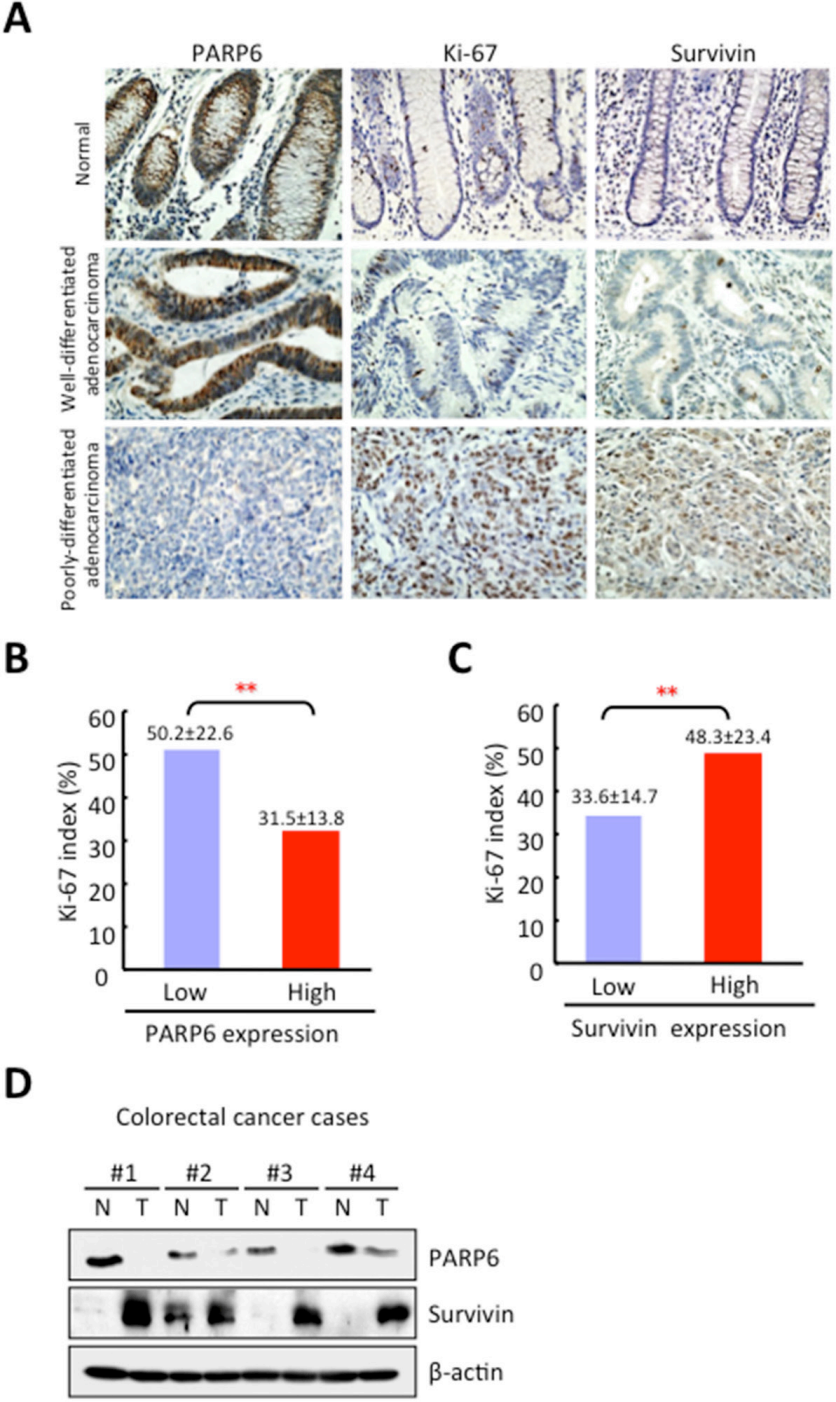
Correlation between PARP6 and Survivin expression in CRC **A.** Expression of PAPR6 and Survivin in 238 CRC cases was examined by immunohistochemistry. Representative images of PARP6, Ki-67 and Survivin in normal colonic mucosa, well differentiated adenocarcinoma and poorly differentiated adenocarcinoma cases. **B.** Correlation between PARP6 and proliferation marker, Ki-67 in CRC cases by immunohistochemistry. Graph shows Ki-67 index in CRC cases with low or high expression of PARP6. **C.** Correlation between PARP6 and Survivin in CRC cases by immunohistochemistry. Graph shows Ki-67 index in CRC cases with low or high expression of Survivin. **D.** The expression of PAPR6 and Survivin were determined in 4 CRC tissues and normal adjacent colorectal tissues by Western blot analysis. β-actin was used as a loading control.

We examined PARP6 expression and its correlation with clinico-pathological findings and proliferation marker, Ki-67 in 20 normal colonic mucosa and 238 CRC samples by immunohistochemistry. Cytoplasmic PARP6 exhibited strong staining in all normal colonic mucosa, but 116 of 238 (48.7%) CRC cases exhibited high expression of PARP6 in the cytoplasm (Figure 4A and Table 1). We observed that the PARP6-positive cells were particularly observed in the cytoplasm of well differentiated adenocarcinoma, but not in poorly differentiated adenocarcinoma (Figure 4A). Low expression of PARP6 was significantly correlated with malignant behaviors including lymph node metastasis, histological differentiation and tumor staging (Table 1). Ki-67 was more frequently expressed in poorly differentiated adenocarcinoma (Figure 4A). Among 238 CRC cases, Ki-67 positivity was higher in PARP6-negative cases, in comparison with PARP6-positive cases (Figure 4B).

**Table 1:** Expression ofPARP6 and Survivin and its correlation with Clinico-pathological features in CRC

Clinico-pathological features	PARP6 expression			Survivin expression		
	Low	High	*p*-value	Low	High	*p*-value
Normal colonic mucosa	20	0		0	20	
Colorectal cancer	122	116		151	87	
Age (years)						
≥50	110	111		142	79	
<50	12	5		9	8	
Sex						
Male	70	70		94	46	
Female	52	46		57	41	
Tumor size (mm)						
≥50	51	48		60	39	
<50	71	68		91	48	
Histological differentiation						
Por/Muc	42	6	*<0.01*	21	27	*<0.01*
W/M	80	110		130	60	
Lymph node metastasis						
Negative	50	75	*<0.01*	99	26	*<0.01*
Positive	72	41		52	61	
Lymphatic invasion						
Negative	10	16		21	5	
Positive	112	100		130	82	
Venous invasion						
Negative	28	29		42	15	
Positive	94	87		109	72	
Tumor stage						
BC	82	106	*<0.01*	135	53	*<0.01*
D	40	10		16	34	

In this study, we found downregulation of Survivin in FL-PARP6 overexpressing CRC cells. Moreover, we previously found that Survivin overexpression is correlated with malignant behavior including cell proliferation activity in CRC [23, 25, 26]. Therefore, we examined the correlation between PARP6 and Survivin in the serial sections from the same CRC cases by immunohistochemical analysis. In 238 CRC cases, high expression of Survivin was observed in 87 (36.5%) cases (Table 1). As expected, Survivin expression was significantly correlated with malignant behaviors including lymph node metastasis, histological differentiation and tumor staging (Table 1). High expression of Survivin was observed in both nucleus and cytoplasm of CRC cells, but Survivin expression was not observed in normal colonic mucosa cases (Figure 4A and Table 1). The expression patterns of the Ki-67 and Survivin appeared to be similar in CRC cases (Figure 4A). The frequency of Ki-67-positive cells was higher in the Survivin-positive cases than in the Survivin-negative cases (Figure 4C). Interestingly, PARP6 expression was inversely correlated with Survivin expression in CRC cases (Figure 4A and Table 2).

**Table 2:** Correlation between PARP6 and Survivin expression in CRC

	PARP6 expression		Total	*P*-value
	Low	High		
Survivin expression				
Low	61	90	151	<0.01
High	61	26	87	
Total	122	116		

We compared the expression pattern of PARP6 and Survivin with clinico-pathological findings in CRC cases. CRC cases with high expression of PARP6 and low expression of Survivin significantly decreased the lymph node metastasis, histological differentiation and delayed tumor progression (Table 3). On the other hand, CRC cases with low expression of PARP6 and high expression of Survivin significantly increased lymph node metastasis, histological differentiation and promoted tumor progression (Table 3).

**Table 3:** Combination of PARP6 and Survivin expression and its correlation with Clinico-pathological features in CRC

PARP6 expression Survivin expression	Low High	High High	Low Low	High Low	*P*-value
Total	61	26	61	90	
Age (years)					
≥50	54	25	56	86	
<50	7	1	5	4	
Sex					
Male	30	16	40	54	
Female	31	10	21	36	
Tumor size (mm)					
≥50	24	15	27	33	
<50	37	11	34	58	
Histological differentiation					
Por/Muc	25	2	17	4	<0.01
W/M	36	24	44	86	
Lymph node metastasis					
Negative	15	11	35	64	<0.01
Positive	46	15	26	26	
Lymphatic invasion					
Negative	3	2	7	14	
Positive	58	24	54	76	
Venous invasion					
Negative	9	6	19	23	
Positive	52	20	42	67	
Tumor stage					
BC	34	19	48	87	<0.01
D	27	7	13	3	

To confirm the inverse correlation between PARP6 and Survivin, we examined the expression of PARP6 and Survivin in 4 cases of CRC tissues and normal adjacent colorectal tissues by Western blotting. The expression levels of the PARP6 protein in the normal adjacent colon tissues were higher than those in the CRC tissues (Figure 4D). However, Survivin displayed an opposite expression pattern in the CRC and adjacent colon tissues (Figure 4D).

### Survival analysis

**Figure 5:**
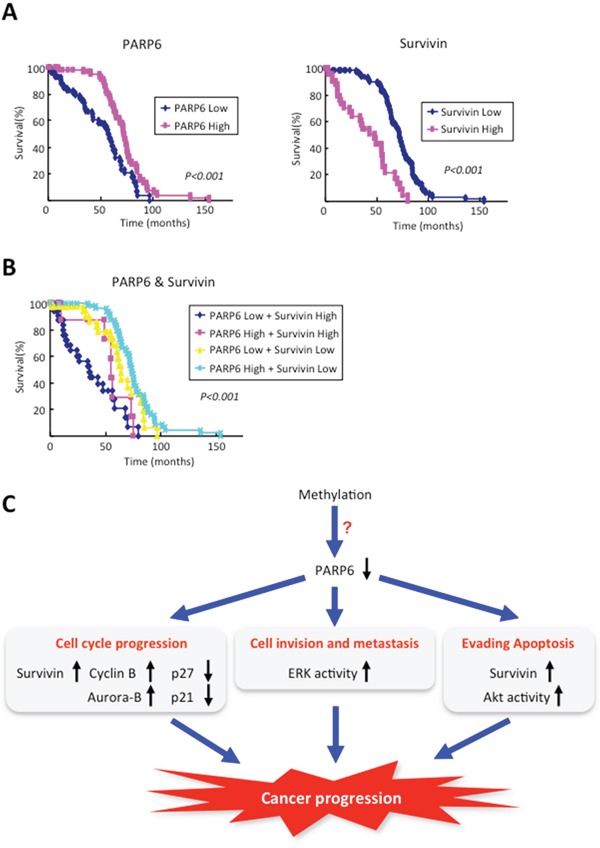
The prognostic value of PAPR6 and Survivin in CRC cases Kaplan–Meier curves with a univariate analysis (log-rank) for colorectal cancer according to the expression of PAPR6 and Survivin. **A.** Left graph shows Kaplan–Meier curves for colorectal cancer according to the expression of PAPR6. The prognosis for patients with high PAPR6 expression (n=66) was better than the prognosis for patients with low PAPR6 expression (n=79) (*P*<0.001). Right graph shows Kaplan–Meier curves for colorectal cancer according to the expression of Survivin. The prognosis for patients with high Survivin expression (n=49) was poorer than the prognosis for patients with low Survivin expression (n=96) (*P*<0.001). **B.** The survival analysis of the simultaneous expression of PARP6 and Survivin. The prognosis for patients with both high PARP6 expression and low Survivin expression is the best (n=54), and the patients with the worst prognosis exhibited both low PARP6 expression and high Survivin expression (n=37). The prognoses of the other two groups were in the middle (n=54) (*P*<0.001). **C.** Schematic model of the role of PARP6 in CRC progression.

To examine the survival rate of 145 CRC patients with high or low expression of PARP6 and/or Survivin, we used the Kaplan-Meier analysis. The 5-year survival rate of the CRC cases with high PARP6 expression was significantly higher than those with low expression (Figure 5A). However, the CRC cases with high Survivin expression had a lower 5-year survival rate compared to the cases with low Survivin expression cases (Figure 5A). Moreover, the 5-year survival rate of CRC cases with both high PARP6 expression and low Survivin expression is the highest among the CRC cases with different pattern of PARP6 and Survivin expression (Figure 5B). On the other hand, CRC cases with low expression of PARP6 and high expression of Survivin showed the lowest 5-year survival rate (Figure 5B).

## DISCUSSION

PARPs are DNA-dependent nuclear enzymes that alter protein-protein and protein-DNA interactions by transferring negatively charged ADP-ribose moieties from cellular nicotinamide-adenine-dinucleotide (NAD) to a variety of protein substrates [7, 12, 29]. So far, 17 members of the PARP family have been identified, and the PARP family is involved in the development of various diseases including cancer [9, 10]. PARP6 is a new member of the PARP family, and the roles and underlying mechanisms of PARP6 in CRC remain unknown. In this study, we demonstrated that PARP6 has key roles in suppressing CRC progression both *in vitro* and *in vivo*. Indeed, reduced expression of PARP6 protein was observed in CRC tissues, in comparison with normal adjacent colon tissues by immunohistochemical analysis and Western blot analysis. Supportively, mRNA level of PARP6 in CRC tissues was much lower than that in normal colon tissues by microarray analysis using Oncomine™ (data not shown). Importantly, CRC cases with PARP6 expression showed better prognosis. Recently, it has been shown that hypermethylation of PARP6 was found in hepatoblastoma and was well correlated with poor prognosis (Meeting abstract in Nihon Geka Gakkai Zasshi 115, p308, 2014 [in Japanese]). Therefore, we suggest that downregulation of PARP6 in CRC may be caused by hypermethylation of its promoter region (Figure 5C).

Survivin is known as a bifunctional protein involved in suppression of apoptosis and mitosis. It is well know that high expression of Survivin is widely observed in various human cancers and associated with a poor outcome [30–32]. Moreover, Survivin overexpression is associated with a poor outcome in CRC patients [23, 25, 26]. Interestingly, we found that PARP6 expression was negatively correlated with Survivin expression in CRC cases and tissues. Moreover, ectopic overexpression of PARP6 downregulated Survivin proteinin CRC cells. Importantly, CRC cases with both low expression of PARP6 and high expression of Survivin showed poor prognosis among the CRC cases with different pattern of PARP6 and Survivin expression. Although the mechanism of downregulation of Survivin by PARP6 is still unclear, our findings suggest that the PARP6 expression in combination with Survivin expression can be useful for prognostic marker in CRC patients.

Here we demonstrated that the PARP6 inhibited cell proliferation, colony formation, invasion and migration, and promotes apoptosis via its catalytic domain in C-terminus in CRC cells. Interestingly, ectopic expression of PARP6 decreased the expression of Survivin, Cyclin B, Aurora-B, Bcl-2, Bcl-XL and increased the expression of p21, p27 and Bax. Moreover, ectopic PARP6 overexpression inhibited the Akt and ERK signaling pathways. Although it is unclear whether alteration of these proteins is induced by PARP6 in a direct or indirect manner, catalytic domain in C-terminus of PARP6 is essential for this phenomenon. ERK and Akt signaling pathways are upstream of the regulation of cell cycle and apoptosis. Therefore, to know the tumor suppressive function of PARP6, the mechanism of suppression of ERK and Akt activity by PARP6 are required to be clarified.

In conclusion, we found that PARP6 has tumor suppressive roles in CRC via inhibition of cell growth, migration and invasion and promotion of apoptosis (Figure 5C). We believe that PARP6 may be useful for diagnostic marker and therapeutic target in clinical application for CRC.

## MATERIALS AND METHODS

### Cell lines and cell culture

The human CRC cell line, SW480, was purchased from American Type Culture Collection (ATCC). The cells were cultured in Dulbecco's Modified Eagle's medium (DMEM) with 10% fetal bovine serum (FBS), penicillin (50 U/ml) and streptomycin (50 g/ml) and maintained at 37°C in a humidified atmosphere of 5% CO_2_.

### Plasmid construction and transfection

A full-length cDNA clone encoding PARP6 and C-terminal deletion mutant (deletion of 436-630 amino acid residues) PARP6 were subcloned into a pEGFP vector (pEGFP-FL-PARP6 and pEGFP-ΔC-PARP6). The pEGFP-FL-PARP6 plasmid, pEGFP-ΔC-PARP6 plasmid or the empty vector was introduced into the SW480 cells using Lipofectamine 2000. The stable clones were obtained by G418 selection (500 mg/ml, Gibco) in the culture medium for 2 weeks. In this study, we used pool stable clones.

### MTT assay

A 3-(4, 5-dimethylthiazol-2-yl)-2, 5-diphenyl tetrazolium (MTT) assay was used to assess cell proliferation. The SW480 cells were transfected with the p-EGFP-empty, p-EGFP-FL-PARP6 or pEGFP-ΔC-PARP6 plasmid. After 48 h, the transfected cells (1500 cells / well) were replated in 96-well plates. The MTT assay was performed to test the cell viability at 24 h, 48 h and 72 h, and the absorbance was measured at 490 nm on microplate reader.

### Colony formation assay

The SW480 cells were transfected with the p-EGFP-empty, p-EGFP-FL-PARP6 or pEGFP-ΔC-PARP6 plasmid. After 48 h, the transfected cells were plated in 6 cm dishes at a density of 500 cells per dish and grown in complete medium for 2 weeks. When the clone was visible with the naked eye, of the culture was terminated, and the colonies were fixed for 10 minutes with methanol, stained with crystal violet for 15 minutes, and counted under an inverted microscope.

### Wound healing assay

For the wounding healing experiment, the transfected cells were planted in 6-well plates and allowed to grow to complete confluence. Next, the cells were wounded by scratching the cell monolayer with a sterile plastic 200 μl micropipette tip to create a cleared area. Subsequently, the wounded cell layer was rinsed with fresh medium to remove the loose cells. Then, serum-free medium was added to the well. After incubation at 37°C for 24 h, we captured digital phase-contrast images using an inverted microscope to monitor the wound healing process. The time point immediately following scratch wounding was set at 100%. Finally, we measured the widths of the wounds on the images and calculated the mean percentage of the total distances of the wound areas.

### Cell invasion assay

Invasion assays were performed in 24-well Transwell chambers (BD Biosciences, Bedford, MA, USA) containing polycarbonate filters with 8 μm pores covered with Matrigel (BD Biosciences). The lower compartment contained 0.5 ml of 10% fetal bovine serum medium. Approximately 1.5×10^5^ transfected cells were resuspended in 200 μl of serum-free medium and placed in the upper chamber. After incubation for 24 h at 37°C in 5% CO_2_, the non-invaded cells on the upper surface were removed with a cotton swab, and the cells that had migrated to the lower surface of the membrane were fixed with methanol and stained with hematoxylin for 10 min each. Then, the filters with migrated cells attached to the lower surface were washed three times with PBS. We performed the assay 3 times and 5 randomly selected fields from each membrane were counted under a light microscope at x100 magnification.

### Western blot analysis

The colon tissue samples and cell line were incubated with RIPA buffer on ice before being subjected to the Western blot analysis. The protein concentration was detected by the Bradford method, with BSA (Sigma-Aldrich) as the standard. Equal amounts of the cell and tissue extracts (40 μg) were subjected to SDS-PAGE and transferred to nitrocellulose membranes (Bio-Rad) for antibody blotting. The membrane was then blocked and incubated with the primary and secondary antibodies. The primary antibodies aginst PARP6 (HPA026991, Sigma-Aldrich), Survivin (NB500-201, Novus Biologicals, Littleton, CO), Aurora-B (Transduction Laboratories), Cyclin B1 (Cell Signaling technology), Caspase 3 (Cell Signaling technology), cleaved Caspase-3 (Cell Signaling technology), Bcl-2 (Cell Signaling technology), Akt (Cell Signaling technology), phospho-Akt (Cell Signaling technology), p38 (Cell Signaling technology), phospho-p38 (Cell Signaling technology), β-actin (Cell Signaling technology), p21 (Santa Cruz Biotechnology), p27 (Santa Cruz Biotechnology), Bax (Santa Cruz Biotechnology), Bcl-XL (Santa Cruz Biotechnology), ERK (Santa Cruz Biotechnology), and phospho-ERK (Santa Cruz Biotechnology) were used.

### Xenograft

Ten male nude mice (5-6 weeks old) were purchased from the Animal Experiment Center of Guilin Medical University. All of the animal experiments were approved by the Guilin Medical University Animal Care and Use Committee. We randomly divided the nude mice into two groups; Control group (p-EGFP-empty vector transfectant cells) and PARP6 group (p-EGFP-FL-PARP6 transfectant cells). The stable pool clones of p-EGFP-empty vector and p-EGFP-FL-PARP6 were collected in the logarithmic growth phase. After washing with PBS, the cells were suspended in serum-free medium. Subsequently, 200 μl of the cell suspension (containing 2×10^7^ cells) was subcutaneously injected into the right groin area of the mice. After the tumors had formed, the mice were observed for tumor growth at 7, 14, 21 and 28 days. The tumor volumes were calculated with the following formula: tumor volume (mm^3^) =0.5 × length (mm) × width^2^ (square mm). After 28 days, the nude mice were euthanized. The tumors were excised and weighed to record the wet tumor weight.

### Patient and tissue samples

The CRC and normal tissues were obtained from 4 patients who underwent surgery at the affiliated hospital of Guilin Medical University. Paraffin-embedded advanced CRC specimens from a total of 238 cases (140 males and 98 females), including 113 cases with lymph node metastasis, were selected from the pathological files of Hiroshima University and Guilin University Hospital (1990-2012). All samples were obtained after approval by the Ethics Committees of Guilin Medical University and Hiroshima University. The age of patients ranged from 37 to 85 years (mean, 62.8 years). Histologically, 190 cases were classified as well and moderately differentiated CRC, while 48 cases were classified as poorly differentiated CRC. The patients' clinical characteristics, including age, sex, tumor size, tumor differentiation, lymph node metastasis, vascular invasion and tumor stage, were gathered from the patients' surgical records. Ninety-three patients who were lost to follow-up were excluded from this study; 145 CRC patients were subjected to a further survival analysis. In addition, 20 sample of normal colonic mucosa were examined as controls. The tumors from each patient were formalin-fixed and cut into parallel 4-5 mm sections. Informed consent was obtained from all subjects.

### Immunohistochemical staining

The sections were incubated with a polyclonal anti-Survivin antibody (NB500-201, Novus Biologicals, Littleton, CO, 1:1000) and an anti-PARP6 antibody (HPA026991, Sigma-Aldrich, 1:500). In addition, to analyze the cell proliferation and correlate it with PARP6 and Survivin expression, we examined Ki-67 expression using an anti-Ki-67 monoclonal antibody (MIB-1, Dako). The sections were incubated with the primary antibodies overnight at 4°C after antigen retrieval by microwave treatment in citrate buffer (pH 6.0). The antibodies were detected with the streptavidin-biotin peroxidase system (Universal LSAB™2 kit, Dako, Kyoto, Japan). The immunohistochemistry grade was defined as - to +++ according to the number of stained cells and the intensity of the reaction in the individual cells. The grades were defined as follows: -, the majority of the cells were not positive; +, 5-20% of the tumor cells showed weak to moderate immunoreactivity; ++, 20-50% of the tumor cells showed moderate immunoreactivity; +++, over 50% of the tumor cells showed intense immunoreactivity. The PARP6- and Survivin-positive cases were graded as ++ or +++, and the negative cases were graded as - or +. A labeling index of the percentage of Ki-67-positive cells was determined by examining at least 1000 tumor cells in five representative areas at ×200 magnification.

### Statistical analysis

The SPSS software package was used for the statistical analyses. The χ^2^ test was used to compare the data between the groups. The survival analysis was conducted using the Kaplan-Meier method and the survival characteristics and was compared using log-rank tests. A *P*-value <0.05 was required for significance.
